# Obstruction of the Intestine and Laparotomy in a Dog

**Published:** 1897-03

**Authors:** 


Obstruction of the Intestine and Laparotomy. A watchdog that had been playing with a small rubber ball presented, several days afterward, all the symptoms of an intestinal obstruction on which emetics or purgatives had no effect. The subject was brought to the veterinary college and anæsthetized by Dastre and Morat's mixture, and shortly after administering the chloroform solution the animal presented symptoms of syncope; but after having performed artificial respiration for a short time the respirations became natural and the operation was proceeded with. An enlargement was felt on the right side, just anterior and below the kidney. An incision was made on the abdominal wall, and the intestine was found very large, congested, and hard. An opening was made in the intestine about four centimetres long, and a small playball was taken from it. To assist in making the suture in the intestinal wall a piece of bread was moulded the shape and circumference of the intestine and put in the opening, which greatly assisted the suture being properly made; the peritoneum and abdominal walls were stitched, and lastly the skin, and an antiseptic was applied to the region. The day following the operation the animal was put under a special regimen, externally having the application of the antiseptic and internally small doses of benzonaphthol. The operation was followed by a quick recovery. It goes without saying that in an operation of laparotomy all antiseptic precautions must be taken.—*Journal de Médecine Vétérinaire et de Zootechnie.*

